# Long-term Outcome of Kidney Transplantation in 6 Patients With Autoimmune Polyendocrinopathy-candidiasis-ectodermal Dystrophy

**DOI:** 10.1097/TP.0000000000003993

**Published:** 2021-11-24

**Authors:** Saila Laakso, Henna Kaijansinkko, Anne Räisänen-Sokolowski, Timo Jahnukainen, Janne Kataja, Outi Mäkitie, Ilkka Helanterä, Hannu Jalanko

**Affiliations:** 1 Children’s Hospital and Pediatric Research Center, Helsinki University Hospital and University of Helsinki, Helsinki, Finland.; 2 Research Program for Clinical and Molecular Metabolism, Faculty of Medicine, University of Helsinki, Helsinki, Finland.; 3 Folkhälsan Research Center, Helsinki, Finland.; 4 Department of Pediatrics and Adolescent Medicine, Tampere University Hospital, Tampere, Finland.; 5 Department of Pathology, Helsinki University Hospital and University of Helsinki, Helsinki, Finland.; 6 Department of Pediatrics and Adolescent Medicine, Turku University Hospital, Turku, Finland.; 7 Department of Molecular Medicine and Surgery, Karolinska Institutet and Clinical Genetics, Karolinska University Hospital, Stockholm, Sweden.; 8 Department of Transplantation and Liver Surgery, Helsinki University Hospital and University of Helsinki, Helsinki, Finland.

Autoimmune polyendocrinopathy-candidiasis-ectodermal dystrophy (APECED; autoimmune polyendocrine syndrome type 1; OMIM 240300) is caused by mutations in the autoimmune regulator gene (*AIRE*) that lead to failure of negative selection of autoreactive T cells and to impaired function of regulatory T cells.^1^ Hypoparathyroidism and primary adrenal insufficiency are the most common early endocrinopathies, but new autoimmune manifestations may appear throughout life. Tubulointerstitial nephritis (TIN) is a severe manifestation of APECED. Kidney biopsies reveal tubular basement membrane (TBM) deposits along with the classic changes of TIN.^2,3^ Other kidney-related problems described are nephrocalcinosis, nephrolithiasis, and distal renal tubular acidosis.^3^ There are two published case reports of kidney transplantation (KT) in APECED.^2,4^

We report 6 patients (6.3%) of the Finnish APECED cohort (n = 95) who underwent in total eight KTs at the age of 12–49 y during 1983–2016 (Table 1). TIN was diagnosed 1.3–10.2 y earlier. One patient was given steroids for TIN, and another received 2 doses of rituximab and mycophenolate mofetil for a year, without response in either case.

**TABLE 1.** Kidney transplantations and clinical characteristics in patients with tubulointerstitial nephritis due to APECED

Patient number	Nro 1	Nro 2	Nro 3	Nro 4	Nro 5, first	Nro 5, second	Nro 6, first	Nro 6, second
Gender	Female	Male	Female	Female	Female		Female	
AIRE genotype	c.769C>T/c.769C>T	c.967–979del13bp/x	c.769C>T/c.769C>T	c.769C>T/c.769C>T	c.769C>T/c.769C>T		c.769C>T/c.769C>T	
TIN to KT (y)	4.6	2.4	3.7	7.6	1.3		10.2	
Dialysis to KT (y)	0.5	0.4	0.3	1.8	1.3		4.7	
Age at KT^a^	15–20	10–15	20–25	25–30	20–25	50–55	45–50	50–55
Year at KT	2016 (deceased donor)	2005 (deceased donor)	1989 (deceased donor)	2014 (deceased donor)	1983 (living donor)	2015 (deceased donor)	2012 (deceased donor)	2017 (deceased donor)
IS	Basiliximab + CsA + MMF + steroids	CsA+ Aza+ steroids	CsA + Aza+ steroids	CsA+ MMF + steroids	CsA + Aza+ steroids	Tac + MMF + steroids	CsA + MMF + steroids	Basiliximab + Tac + MMF + steroids
Early outcome	Delayed graft function	Early graft function	Early graft function	Early graft function	Early graft function	Early graft function	Primary nonfunction	Delayed graft function
Rejections								
Biopsy	TCMR (Gr1A) at 8. days post-KT; TIN relapse at 1.5 y post-KT, abundant IgG, C3, and C4d at tubular membrane.	Borderline TCMR at 3 mo. Mild lymphocytic interstitial inflammation and tubulitis.	TCMR (Gr1A) 29 days post-KT.	No rejections. Interstitial fibrosis, no tubulitis, C4d negative.	Normal biopsies, no sign of rejection or TIN.	TCMR (Gr1A) at 10 days post-KT.	TCMR (Gr1A) 11 days post-KT.	Normal biopsies, no sign of rejection or TIN.
Treatment	Reactive to MP pulses; Reactive to anti-CD20 therapy.	Reactive to MP pulses and conversion to Tac.	Reactive to MP pulses.			Reactive to MP pulses.	Reactive to MP pulses.	
e/mGFR at 1 y	34 mL/min	86 mL/min	80 mL/min	43 mL/min	27 mL/min	26 mL/min	No graft function	67 mL/min
Pre; Post-KT PRA	0/1; 0/1	0/0; 0/53	NA; NA	8/0; NA	NA; 20/49	20/49; 20/49	1/0; 100/99	95/99; NA
Pre; Post-KT DSA	No; DR52	No; DQ7	NA; NA	No; No	NA; A1	No; No	No; A24, B27, DR15, DQ6	No; NA
Pre; Post-KT MFI	NA; 1060	NA; 5224	NA; NA	NA; NA	NA; 2993	NA; NA	NA; 9130, 6127, 8355, 10328	NA; NA
Persistent DSA	No	Yes	NA	NA	Yes	NA	Yes	NA
De novo DSA	Yes	Yes	NA	NA	No	NA	Yes	NA
APECED manifestations before KT	CMC, rash with fever, HP, iridocyclitis, PAI	CMC, hypothyroidism, PAI, GH, rash with fever, AIHA,^b^ hepatitis,^b^ exocrine pancreas insufficiency	HP, CMC, PAI, alopecia, POI	HP, POI, glaucoma, hypothyroidism	CMC, HP		HP, CMC, PAI, POI, DM	
Findings during the follow-up after KT								
New APECED manifestations	POI	–	Epithelial carcinoma of tongue, hyposplenia, hypothyroidism	PAI	PAI, alopecia, atrophic gastritis		–	
CMC	Prophylactic medication	Recurrent in mouth, esophagus	Recurrent in mouth	Prophylactic medication	Recurrent in mouth, vagina		Prophylactic medication	
Virus	–	Warts, varicella zoster	–	CMV	CMV		–	
Infections requiring hospitalization	Mastoiditis; Pyelonephritis *Proteus mirabilis*^c^	–	Pneumonia *Pneumocystis carinii; Campylobacter jejuni*	*Escherichia coli* pyelonephritis; Cholecystitis, peritonitis *and E. coli* septicemia.		Pneumonia, *Enterococcus faecalis* pyelonephritis; *Staphylococcus aureus* septicemia		
Long-term outcome	At the age of 19 y alive with functioning graft (mGFR, 39 mL/min)	Deceased at 19 y with a functioning graft	At the age of 54 y alive with functioning graft (eGFR, 71 mL/min)	At the age of 33 y alive with functioning graft (eGFR, 36 mL/min)	Return to dialysis in 2013	Deceased at 58 y with a functioning graft (eGFR, 25 mL/min)	Graft removed 1 y post-KT	Deceased at 56 y with a functioning graft (eGFR, 70 mL/min)

^a^Age is presented in categories to protect patient anonymity.

^b^Transient and not requiring long-term medication.

^c^Treated with regular immunoglobulin infusions.

AIHA, autoimmune hemolytic anemia; AIRE, autoimmune regulator gene; APECED, autoimmune polyendocrinopathy-candidiasis-ectodermal dystrophy; Aza, azathioprine; C4d, complement C4 activation product C4d; CD22, cluster of differentiation-22; CKD-EPI, chronic kidney disease epidemiology collaboration; CMC, chronic mucocutaneous candidiasis; CMV, cytomegalovirus; Cr, creatinine; CsA, cyclosporine; DM, diabetes mellitus type I; DQ, HLA-DQ antigen; DSA, donor-specific antibody; eGFR, estimated GFR with CKD-EPI equation; GFR, glomerular filtration rate; GH, growth hormone deficiency; Gr1A, gradus 1A; HP, hypoparathyroidism; IS, immunosuppressive treatment; KT, kidney transplantation; MFI, mean fluoresce intensity; mGFR, measured GFR with Cr-EDTA- clearance; MMF, mycophenolate mofetil; MP, methylprednisolone; NA, not available; Nro, number; PAI, primary adrenal insufficiency; POI, primary ovarian insufficiency; PRA, panel reactive antibody; Tac, tacrolimus; TCMR, T-cell-mediated rejection; TIN, tubulointerstitial nephritis.

Five of the 6 patients received a deceased donor transplant (crossmatch negative, HLA-mismatch 0/6-3/6) and one living-related donor transplant. One allograft showed primary nonfunction and was removed a year later, and 2 delayed graft function with early recovery. Altogether 2 patients needed re-KT (Table 1). Immunosuppressive triple-medication reflected the changes in treatment protocols over the past decades. Kidney biopsies revealed an acute cellular rejection during the first months after KT in 5 patients. These responded to methylprednisolone pulses. One patient developed TIN recurrence 3 mo after KT, with intense IgG and C3 stainings in TBM. The complement C4 activation product C4d staining was positive in TBM but negative in peritubular capillaries. Repeated rituximab infusions led to recovery 2 y after KT. Differential diagnosis between TIN and rejection in patients with APECED is demanding and should be based on the clinical and biopsy findings as well as the response to immunosuppressive therapy.

All 6 patients needed continuous medications for the APECED manifestations diagnosed preoperatively. Two of the 5 patients with hypoparathyroidism developed severe hypocalcemia perioperatively. During the follow-up, 4 patients experienced severe electrolyte imbalances mainly associated with dehydration and infections. All 5 patients with adrenal insufficiency needed daily hydrocortisone substitution either in combination with methylprednisolone or alone, and 4 of them used mineralocorticoid substitution. Potassium supplementation was common after KT. Electrolyte disturbances are often associated with transient worsening of kidney function.

The incidence of candidiasis was similar to that observed before KT. Episodes of pneumocystis pneumonia, cytomegalovirus infection, bacterial septicemia, pyelonephritis, and peritonitis were successfully treated. No one developed BK pyelomavirus nephritis or post-transplantation lymphoproliferative disease. One patient experienced epithelial carcinoma of the tongue 13 y after KT that is a known complication of APECED.^5^

The overall transplant survival was good. Three patients deceased during the follow-up for causes unrelated to KT with a quite normal allograft function (Table 1). The longest graft survival was 31 y. In conclusion, KT is a viable treatment modality for APECED patients with end-stage kidney disease, but the patients require careful follow-up due to high risk of complications.
